# Crystal structure of 1′-(2-methyl­prop­yl)-2,3-di­hydro­spiro­[1-benzo­thio­pyran-4,4′-imidazolidine]-2′,5′-dione

**DOI:** 10.1107/S1600536814018030

**Published:** 2014-08-23

**Authors:** Cynthia E. Theodore, S. Naveen, S. B. Benaka Prasad, M. Madaiah, C. S. Ananda Kumar, N. K. Lokanath

**Affiliations:** a Department of Chemistry, School of Engineering and Technology, Jain University, Bangalore 562 112, India; b Institution of Excellence, University of Mysore, Manasagangotri, Mysore 570 006, India; c Department of Studies in Chemistry, University of Mysore, Manasagangotri, Mysore 570 006, India; d Department of Nanotechnology, Center for Post Graduate Studies, Visveswaraya Technological University, Bangalore 560 018, India; e Department of Studies in Physics, University of Mysore, Manasagangotri, Mysore m570 006, India

**Keywords:** crystal structure, hydantoin compounds, hydrogen bonding, spiro­[1-benzo­thio­pyran-4,4′-imidazolidine]

## Abstract

In the title compound, C_15_H_18_N_2_O_2_S, the 2,3-di­hydro-1-benzo­thio­pyran ring adopts a sofa conformation and the hydantoin ring is twisted with respect to the benzene ring at 78.73 (17)°. In the crystal, pairs of N—H⋯O hydrogen bonds link the mol­ecules into inversion dimers.

## Related literature   

For background and applications of hydantoin compounds, see: Nefzi *et al.* (2002 ▶); Park & Kurth (2000 ▶); Manjunath *et al.* (2012 ▶); Hussein *et al.* (2014 ▶). For related structures, see: Manjunath *et al.* (2011 ▶); Hussein *et al.* (2014 ▶).
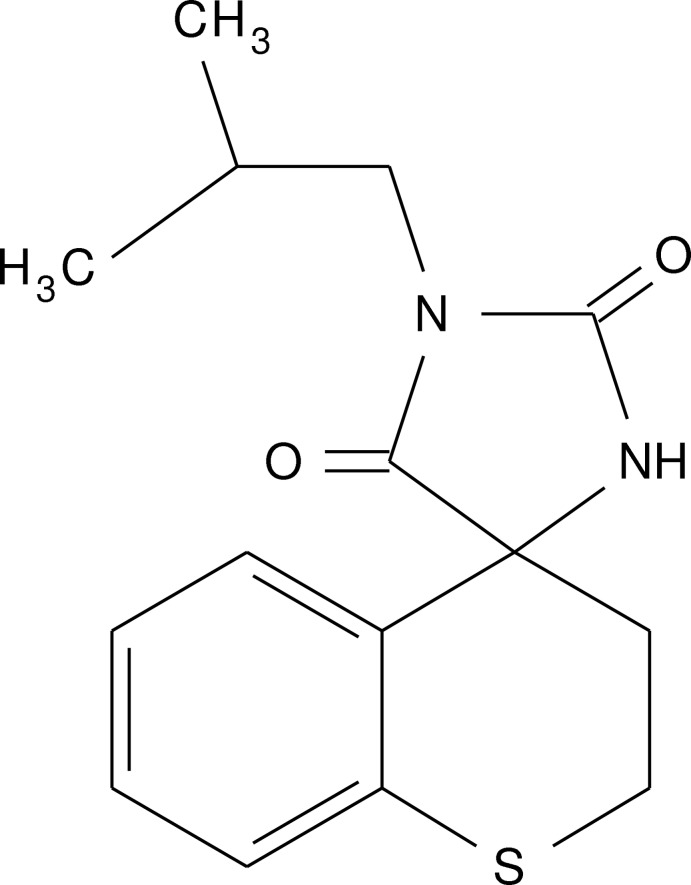


## Experimental   

### Crystal data  

C_15_H_18_N_2_O_2_S
*M* *_r_* = 290.38
Monoclinic, 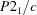

*a* = 13.279 (3) Å
*b* = 9.939 (3) Å
*c* = 13.264 (3) Å
β = 118.56 (1)°
*V* = 1537.6 (7) Å^3^
*Z* = 4
Cu *K*α radiation
μ = 1.90 mm^−1^
*T* = 296 K
0.20 × 0.15 × 0.15 mm

### Data collection  

Bruker X8 Proteum diffractometer
Absorption correction: multi-scan (*SADABS*; Bruker, 2013 ▶) *T*_min_ = 0.747, *T*_max_ = 0.753
5024 measured reflections
2397 independent reflections
1959 reflections with *I* > 2σ(*I*)
*R*_int_ = 0.067

### Refinement  

*R*[*F* ^2^ > 2σ(*F* ^2^)] = 0.066
*wR*(*F* ^2^) = 0.195
*S* = 1.06
2397 reflections
184 parameters
H-atom parameters constrained
Δρ_max_ = 0.43 e Å^−3^
Δρ_min_ = −0.47 e Å^−3^

### 

Data collection: *APEX2* (Bruker, 2013 ▶); cell refinement: *SAINT* (Bruker, 2013 ▶); data reduction: *SAINT*; program(s) used to solve structure: *SHELXS97* (Sheldrick, 2008 ▶); program(s) used to refine structure: *SHELXL97* (Sheldrick, 2008 ▶); molecular graphics: *PLATON* (Spek, 2009 ▶); software used to prepare material for publication: *PLATON*.

## Supplementary Material

Crystal structure: contains datablock(s) global, I. DOI: 10.1107/S1600536814018030/xu5809sup1.cif

Structure factors: contains datablock(s) I. DOI: 10.1107/S1600536814018030/xu5809Isup2.hkl

Supporting information file. DOI: 10.1107/S1600536814018030/xu5809Isup3.cml

**Fig. 1.**
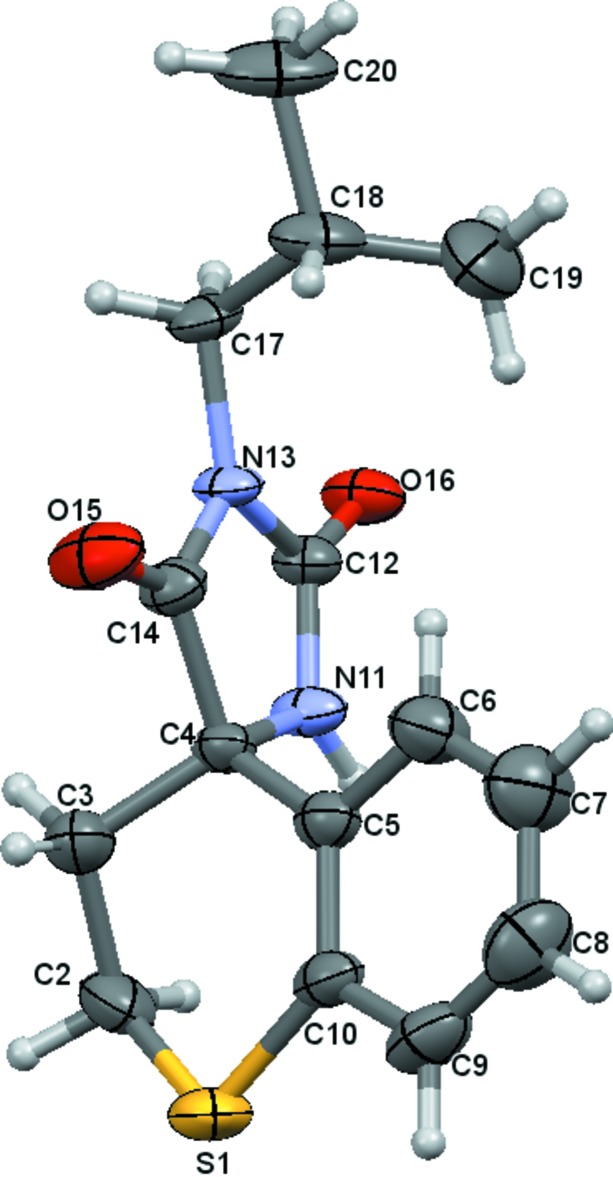
A view of the title molecule, with atom labelling. Displacement ellipsoids are drawn at the 50% probability level.

. DOI: 10.1107/S1600536814018030/xu5809fig1.tif
A view of the title mol­ecule, with atom labelling. Displacement ellipsoids are drawn at the 50% probability level.

**Fig. 2.**
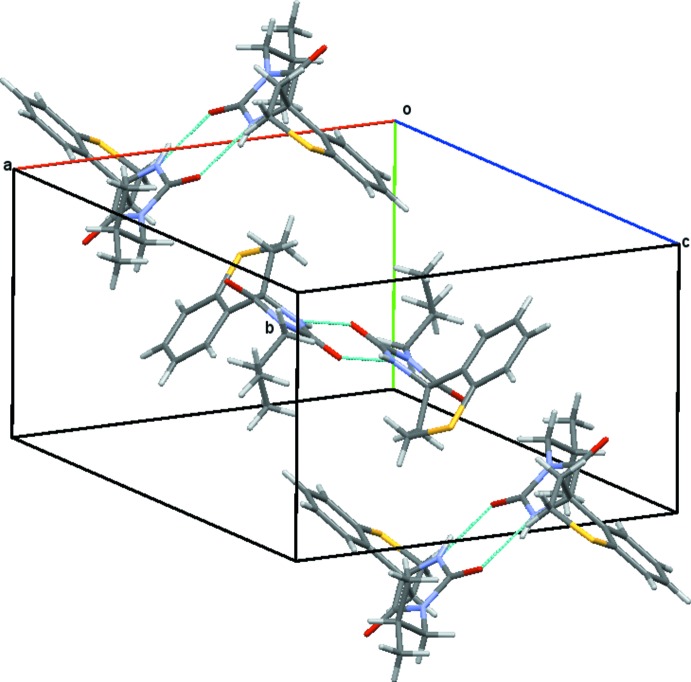
A view of the crystal packing of the title compound showing inverted dimers.

. DOI: 10.1107/S1600536814018030/xu5809fig2.tif
A view of the crystal packing of the title compound showing inverted dimers.

CCDC reference: 1018087

Additional supporting information:  crystallographic information; 3D view; checkCIF report

## Figures and Tables

**Table 1:** Hydrogen-bond geometry (Å, °)

*D*—H⋯*A*	*D*—H	H⋯*A*	*D*⋯*A*	*D*—H⋯*A*
N11—H11⋯O16^i^	0.86	2.03	2.850 (3)	160

Symmetry code: (i) 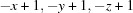.

## supplementary crystallographic information

### S1. Comment

The combinatorial generation of organic compound libraries has emerged as a powerful tool for drug discovery. Small substituted heterocyclic compounds play an important role in the development of biologically active substances by offering a high structural diversity. Among such heterocycles, particularly the hydantoin scaffold opens the possibility of different kinds and degrees of substitution. They have been the focus of attention as a ubiquitous moiety incorporated into compounds with numerous biological activities and therapeutic applications (Nefzi *et al.*, 2002). A variety of combinatorial approaches have been described by which pharmacophoric groups were attached to such a relatively rigid scaffold (Park & Kurth, 2000). Therefore, the chemistry of multiple substituted hydantoins has newly attracted much interest, and traditional approaches have been combined with recently developed strategies. Hence as a part of our ongoing research on hydantoins (Manjunath *et al.*, 2012; Hussein *et al.*, 2014), the synthesis, characterization and the structural work of the title compound was undertaken and herein we report its crystal structure.

The hydantoin ring in the structure is planar within the experimental limits with a maximum deviation of 0.012 (2) Å for N1 atom from the least-squares plane of the hydantoin ring. The N—C bong length values of N11—C12 = 1.349 (3) Å, N13—C12 = 1.400 (4) Å and N13—C14 = 1.359 (2) Å are comparable with the values reported earlier (Manjunath *et al.*, 2011; Hussein *et al.*, 2014). The shortened bond length values can be attributed to the π conjugation in the hydantoin ring. The isobutyl group is twisted out of the plane of the hydantoin ring as indicated by the torsion angle values of -175.6 (3)° and 61.5 (3)° for the atoms N13—C17—C18—C20 and N13—C17—C18—C19 indicating that they are in antiperiplanar and synclinal conformations respectively.

The study of torsion angles, asymmetric parameters and least-squares plane reveals that the 2,3-dihydro-1-benzothiopyran ring in the structure adopts envelope conformation with S1 atom deviating by 0.0851 (14) Å from the least-squares plane. This is confirmed by the puckering amplitude *Q* = 0.519 (3) Å. The hydantoin ring is in a equatorial position with the 2,3-dihydro-1-benzothiopyran ring which is evident by the dihedral angle of 81.15 (15)°. This value is slightly lesser than the value reported earlier (Hussein *et al.*, 2014) for 1-ethyl-2',3'-dihydrospiro[imidazoline-4,1-indene]-2,5-dione. The molecules are interlinked by N—H···O hydrogen bonds to form inverted dimers.

### S2. Experimental

A solution of spiro[1-benzothiopyran-4,4'-imidazolidine]-2',5'-dione (1.0 eq) in *N*,*N*-dimethylformamide was taken, anhydrous K_2_CO_3_ (3.0 eq) was added to the solution and stirred for 10 min. 1-Bromo–2 methyl propane (1–1.1 eq) was then added. The reaction mixture was stirred at room temperature for 8 h and the progress of the reaction was monitored by TLC. Upon completion, the solvent was removed under reduced pressure and the residue was taken in water and extracted with ethyl acetate. Finally water wash was given to the organic layer and dried over anhydrous sodium sulfate. The solvent was evaporated. The crude product was purified by column chromatography using chloroform:methanol (9:1) as an eluent. Single crystals were obtained from slow evaporation of its solvent.

### S3. Refinement

The C-bound hydrogen atom were fixed geometrically (C—H = 0.93–0.97 Å) and allowed to ride on their parent atoms with *U*_iso_(H) = 1.2–1.5*U*_eq_(C). The N-bound H atom was included in the model with N—H = 0.86 Å, and with *U*_iso_(H) = 1.2*U*_eq_(N).

### Crystal data

**Table:**

C_15_H_18_N_2_O_2_S	*Z* = 4
*M**_r_* = 290.38	*F*(000) = 616
Monoclinic, *P*2_1_/*c*	*D*_x_ = 1.254 Mg m^−^^3^
Hall symbol: -P 2ybc	Cu *K*α radiation, λ = 1.54178 Å
*a* = 13.279 (3) Å	µ = 1.90 mm^−^^1^
*b* = 9.939 (3) Å	*T* = 296 K
*c* = 13.264 (3) Å	Block, yellow
β = 118.56 (1)°	0.20 × 0.15 × 0.15 mm
*V* = 1537.6 (7) Å^3^	

### Data collection

**Table:**

Bruker X8 Proteum diffractometer	1959 reflections with *I* > 2σ(*I*)
Detector resolution: 18.4 pixels mm^-1^	*R*_int_ = 0.067
φ and ω scans	θ_max_ = 64.2°, θ_min_ = 3.8°
Absorption correction: multi-scan (*SADABS*; Bruker, 2013)	*h* = −15→14
*T*_min_ = 0.747, *T*_max_ = 0.753	*k* = −8→11
5024 measured reflections	*l* = −11→15
2397 independent reflections	

### Refinement

**Table:**

Refinement on *F*^2^	Secondary atom site location: difference Fourier map
Least-squares matrix: full	Hydrogen site location: inferred from neighbouring sites
*R*[*F*^2^ > 2σ(*F*^2^)] = 0.066	H-atom parameters constrained
*wR*(*F*^2^) = 0.195	*w* = 1/[σ^2^(*F*_o_^2^) + (0.1345*P*)^2^ + 0.3286*P*] where *P* = (*F*_o_^2^ + 2*F*_c_^2^)/3
*S* = 1.06	(Δ/σ)_max_ < 0.001
2397 reflections	Δρ_max_ = 0.43 e Å^−^^3^
184 parameters	Δρ_min_ = −0.47 e Å^−^^3^
0 restraints	Extinction correction: *SHELXL97* (Sheldrick, 2008), FC^*^=KFC[1+0.001XFC^2^Λ^3^/SIN(2Θ)]^-1/4^
Primary atom site location: structure-invariant direct methods	Extinction coefficient: 0.0092 (15)

### Special details

**Table:**

Experimental. H^1^NMR (DMSO, 400 MHz) δ:9.0(s, 1H, –NH), δ:7.2(m, 2H, Ar—H) δ:7.1(m, 2H, Ar—H) δ:3.3(d, 2H, –CH_2_–)δ:3.1(m, 2H, –CH_2_–) δ:2.1(m, 1H, –CH–) δ:0.9(m, 6H, –CH_3_–). Melting point 636.52 K.
Geometry. Bond distances, angles *etc*. have been calculated using the rounded fractional coordinates. All su's are estimated from the variances of the (full) variance-covariance matrix. The cell e.s.d.'s are taken into account in the estimation of distances, angles and torsion angles
Refinement. Refinement on *F*^2^ for ALL reflections except those flagged by the user for potential systematic errors. Weighted *R*-factors *wR* and all goodnesses of fit *S* are based on *F*^2^, conventional *R*-factors *R* are based on *F*, with *F* set to zero for negative *F*^2^. The observed criterion of *F*^2^ > σ(*F*^2^) is used only for calculating -*R*-factor-obs *etc*. and is not relevant to the choice of reflections for refinement. *R*-factors based on *F*^2^ are statistically about twice as large as those based on *F*, and *R*-factors based on ALL data will be even larger.

### Fractional atomic coordinates and isotropic or equivalent isotropic displacement parameters (Å^2^)

**Table:**

	*x*	*y*	*z*	*U*_iso_*/*U*_eq_	
S1	0.50647 (7)	0.34459 (8)	0.13658 (6)	0.0529 (3)	
O15	0.8299 (2)	0.2088 (3)	0.52413 (19)	0.0547 (8)	
O16	0.62741 (16)	0.4706 (2)	0.64774 (16)	0.0400 (6)	
N11	0.59964 (19)	0.4235 (2)	0.46550 (18)	0.0348 (7)	
N13	0.75117 (18)	0.3354 (2)	0.61452 (18)	0.0305 (7)	
C2	0.4715 (3)	0.2679 (3)	0.2388 (3)	0.0490 (10)	
C3	0.5783 (3)	0.2225 (3)	0.3462 (2)	0.0426 (9)	
C4	0.6545 (2)	0.3412 (2)	0.4141 (2)	0.0302 (8)	
C5	0.6935 (2)	0.4273 (3)	0.3446 (2)	0.0343 (8)	
C6	0.7916 (3)	0.5061 (3)	0.4013 (3)	0.0487 (10)	
C7	0.8306 (3)	0.5889 (4)	0.3438 (3)	0.0629 (12)	
C8	0.7704 (4)	0.5939 (4)	0.2254 (3)	0.0637 (12)	
C9	0.6728 (3)	0.5174 (4)	0.1671 (3)	0.0521 (11)	
C10	0.6325 (3)	0.4346 (3)	0.2243 (2)	0.0374 (8)	
C12	0.6543 (2)	0.4167 (3)	0.5811 (2)	0.0299 (7)	
C14	0.7571 (2)	0.2853 (3)	0.5222 (2)	0.0342 (8)	
C17	0.8345 (2)	0.3059 (3)	0.7353 (2)	0.0362 (8)	
C18	0.9406 (3)	0.3929 (4)	0.7806 (2)	0.0475 (10)	
C19	0.9119 (4)	0.5421 (4)	0.7785 (4)	0.0785 (16)	
C20	1.0243 (3)	0.3466 (5)	0.9022 (3)	0.0727 (16)	
H2A	0.42970	0.33190	0.26000	0.0590*	
H2B	0.42210	0.19100	0.20360	0.0590*	
H3A	0.62210	0.16230	0.32440	0.0510*	
H3B	0.55560	0.17280	0.39500	0.0510*	
H6	0.83250	0.50270	0.48100	0.0580*	
H7	0.89640	0.64050	0.38410	0.0760*	
H8	0.79560	0.64880	0.18520	0.0770*	
H9	0.63290	0.52130	0.08730	0.0630*	
H11	0.53910	0.47100	0.42630	0.0420*	
H17A	0.85700	0.21210	0.74160	0.0430*	
H17B	0.79780	0.31930	0.78250	0.0430*	
H18	0.97670	0.37940	0.73190	0.0570*	
H19A	0.86290	0.56990	0.70070	0.1180*	
H19B	0.98140	0.59390	0.81030	0.1180*	
H19C	0.87330	0.55640	0.82310	0.1180*	
H20A	0.99140	0.36250	0.95170	0.1090*	
H20B	1.09470	0.39600	0.92990	0.1090*	
H20C	1.03950	0.25230	0.90150	0.1090*	

### Atomic displacement parameters (Å^2^)

**Table:**

	*U*^11^	*U*^22^	*U*^33^	*U*^12^	*U*^13^	*U*^23^
S1	0.0564 (6)	0.0552 (6)	0.0243 (5)	0.0026 (4)	0.0009 (4)	0.0035 (3)
O15	0.0531 (13)	0.0668 (15)	0.0394 (12)	0.0333 (12)	0.0183 (10)	0.0072 (11)
O16	0.0359 (10)	0.0554 (12)	0.0244 (10)	0.0082 (9)	0.0109 (8)	−0.0034 (8)
N11	0.0302 (11)	0.0462 (13)	0.0214 (11)	0.0122 (10)	0.0071 (9)	0.0028 (9)
N13	0.0249 (11)	0.0396 (12)	0.0168 (11)	0.0062 (9)	0.0017 (9)	0.0031 (9)
C2	0.0441 (17)	0.0490 (17)	0.0367 (16)	−0.0070 (13)	0.0055 (14)	−0.0110 (13)
C3	0.0496 (17)	0.0408 (16)	0.0319 (15)	−0.0034 (13)	0.0151 (13)	0.0008 (12)
C4	0.0347 (14)	0.0328 (14)	0.0190 (13)	0.0056 (10)	0.0096 (11)	−0.0007 (10)
C5	0.0384 (14)	0.0352 (14)	0.0269 (14)	0.0059 (11)	0.0136 (11)	0.0001 (11)
C6	0.0464 (17)	0.0569 (19)	0.0367 (17)	−0.0064 (14)	0.0149 (14)	−0.0048 (14)
C7	0.060 (2)	0.072 (2)	0.059 (2)	−0.0149 (18)	0.0302 (18)	0.0002 (19)
C8	0.070 (2)	0.068 (2)	0.065 (2)	0.0000 (19)	0.042 (2)	0.0157 (19)
C9	0.064 (2)	0.0580 (19)	0.0363 (16)	0.0142 (16)	0.0257 (15)	0.0160 (15)
C10	0.0446 (15)	0.0382 (14)	0.0251 (13)	0.0128 (12)	0.0133 (12)	0.0049 (11)
C12	0.0256 (12)	0.0374 (13)	0.0217 (13)	0.0014 (10)	0.0073 (10)	0.0003 (10)
C14	0.0326 (13)	0.0360 (14)	0.0288 (14)	0.0089 (11)	0.0105 (11)	0.0032 (11)
C17	0.0296 (13)	0.0460 (15)	0.0220 (13)	0.0051 (11)	0.0034 (11)	0.0096 (11)
C18	0.0305 (14)	0.078 (2)	0.0244 (15)	−0.0055 (14)	0.0053 (12)	0.0011 (14)
C19	0.074 (3)	0.063 (2)	0.062 (3)	−0.026 (2)	0.003 (2)	−0.0043 (19)
C20	0.0395 (18)	0.125 (4)	0.0326 (18)	0.005 (2)	0.0003 (15)	0.007 (2)

### Geometric parameters (Å, º)

**Table:**

S1—C2	1.799 (4)	C17—C18	1.511 (5)
S1—C10	1.759 (3)	C18—C19	1.528 (6)
O15—C14	1.221 (4)	C18—C20	1.528 (5)
O16—C12	1.224 (3)	C2—H2A	0.9700
N11—C4	1.463 (4)	C2—H2B	0.9700
N11—C12	1.349 (3)	C3—H3A	0.9700
N13—C12	1.400 (4)	C3—H3B	0.9700
N13—C14	1.359 (3)	C6—H6	0.9300
N13—C17	1.477 (3)	C7—H7	0.9300
N11—H11	0.8600	C8—H8	0.9300
C2—C3	1.521 (5)	C9—H9	0.9300
C3—C4	1.535 (4)	C17—H17A	0.9700
C4—C5	1.519 (4)	C17—H17B	0.9700
C4—C14	1.534 (4)	C18—H18	0.9800
C5—C6	1.393 (5)	C19—H19A	0.9600
C5—C10	1.404 (3)	C19—H19B	0.9600
C6—C7	1.380 (6)	C19—H19C	0.9600
C7—C8	1.381 (5)	C20—H20A	0.9600
C8—C9	1.378 (6)	C20—H20B	0.9600
C9—C10	1.388 (5)	C20—H20C	0.9600
			
C2—S1—C10	102.93 (16)	C3—C2—H2A	109.00
C4—N11—C12	112.6 (2)	C3—C2—H2B	109.00
C12—N13—C14	111.5 (2)	H2A—C2—H2B	108.00
C12—N13—C17	123.8 (2)	C2—C3—H3A	109.00
C14—N13—C17	124.7 (2)	C2—C3—H3B	109.00
C12—N11—H11	124.00	C4—C3—H3A	109.00
C4—N11—H11	124.00	C4—C3—H3B	109.00
S1—C2—C3	111.8 (3)	H3A—C3—H3B	108.00
C2—C3—C4	112.3 (2)	C5—C6—H6	119.00
N11—C4—C3	111.6 (3)	C7—C6—H6	119.00
C3—C4—C5	113.4 (2)	C6—C7—H7	121.00
N11—C4—C5	110.99 (19)	C8—C7—H7	120.00
N11—C4—C14	100.64 (19)	C7—C8—H8	120.00
C5—C4—C14	111.3 (2)	C9—C8—H8	120.00
C3—C4—C14	108.2 (2)	C8—C9—H9	119.00
C4—C5—C10	122.7 (3)	C10—C9—H9	119.00
C4—C5—C6	119.4 (2)	N13—C17—H17A	109.00
C6—C5—C10	117.9 (3)	N13—C17—H17B	109.00
C5—C6—C7	122.6 (3)	C18—C17—H17A	109.00
C6—C7—C8	118.9 (4)	C18—C17—H17B	109.00
C7—C8—C9	119.8 (4)	H17A—C17—H17B	108.00
C8—C9—C10	121.7 (3)	C17—C18—H18	108.00
S1—C10—C9	115.7 (2)	C19—C18—H18	108.00
C5—C10—C9	119.2 (3)	C20—C18—H18	108.00
S1—C10—C5	125.1 (3)	C18—C19—H19A	109.00
N11—C12—N13	107.6 (2)	C18—C19—H19B	109.00
O16—C12—N11	128.0 (3)	C18—C19—H19C	110.00
O16—C12—N13	124.4 (2)	H19A—C19—H19B	109.00
N13—C14—C4	107.6 (2)	H19A—C19—H19C	109.00
O15—C14—N13	126.6 (2)	H19B—C19—H19C	110.00
O15—C14—C4	125.9 (2)	C18—C20—H20A	109.00
N13—C17—C18	113.0 (2)	C18—C20—H20B	109.00
C19—C18—C20	111.2 (3)	C18—C20—H20C	110.00
C17—C18—C19	111.8 (3)	H20A—C20—H20B	109.00
C17—C18—C20	108.5 (3)	H20A—C20—H20C	110.00
S1—C2—H2A	109.00	H20B—C20—H20C	109.00
S1—C2—H2B	109.00		
			
C10—S1—C2—C3	−37.5 (3)	N11—C4—C14—N13	0.2 (3)
C2—S1—C10—C5	7.6 (3)	C14—C4—C5—C10	146.9 (3)
C2—S1—C10—C9	−173.0 (3)	N11—C4—C5—C6	76.0 (3)
C4—N11—C12—N13	2.7 (3)	N11—C4—C5—C10	−101.9 (3)
C4—N11—C12—O16	−177.7 (3)	C3—C4—C5—C6	−157.4 (3)
C12—N11—C4—C5	−119.6 (2)	C3—C4—C5—C10	24.6 (4)
C12—N11—C4—C14	−1.8 (3)	C3—C4—C14—O15	62.5 (4)
C12—N11—C4—C3	112.8 (3)	C3—C4—C14—N13	−117.0 (3)
C17—N13—C12—O16	−0.9 (4)	C5—C4—C14—O15	−62.8 (4)
C14—N13—C12—N11	−2.6 (3)	C5—C4—C14—N13	117.8 (2)
C12—N13—C14—C4	1.4 (3)	C10—C5—C6—C7	−0.6 (5)
C14—N13—C12—O16	177.8 (3)	C4—C5—C10—S1	−1.6 (5)
C12—N13—C17—C18	−99.0 (3)	C4—C5—C6—C7	−178.6 (3)
C14—N13—C17—C18	82.5 (3)	C4—C5—C10—C9	179.0 (3)
C12—N13—C14—O15	−178.0 (3)	C6—C5—C10—S1	−179.6 (3)
C17—N13—C14—C4	−179.9 (2)	C6—C5—C10—C9	1.0 (5)
C17—N13—C12—N11	178.8 (2)	C5—C6—C7—C8	−0.2 (6)
C17—N13—C14—O15	0.7 (5)	C6—C7—C8—C9	0.4 (7)
S1—C2—C3—C4	65.3 (3)	C7—C8—C9—C10	0.1 (7)
C2—C3—C4—C5	−57.9 (4)	C8—C9—C10—C5	−0.8 (6)
C2—C3—C4—C14	178.1 (3)	C8—C9—C10—S1	179.8 (3)
C2—C3—C4—N11	68.3 (3)	N13—C17—C18—C19	61.5 (3)
C14—C4—C5—C6	−35.2 (4)	N13—C17—C18—C20	−175.6 (3)
N11—C4—C14—O15	179.6 (3)		

### Hydrogen-bond geometry (Å, º)

**Table:**

*D*—H···*A*	*D*—H	H···*A*	*D*···*A*	*D*—H···*A*
N11—H11···O16^i^	0.86	2.03	2.850 (3)	160

Symmetry code: (i) −*x*+1, −*y*+1, −*z*+1.
