# Supplementary figures and images for: Crystal structure of 1′-(2-methyl­prop­yl)-2,3-di­hydro­spiro­[1-benzo­thio­pyran-4,4′-imidazolidine]-2′,5′-dione
Source: Acta Crystallogr Sect E Struct Rep Online. 2014 Aug 23;70(Pt 9):o1043–4. doi: 10.1107/S1600536814018030 (PMC4186156; doi:10.1107/S1600536814018030)

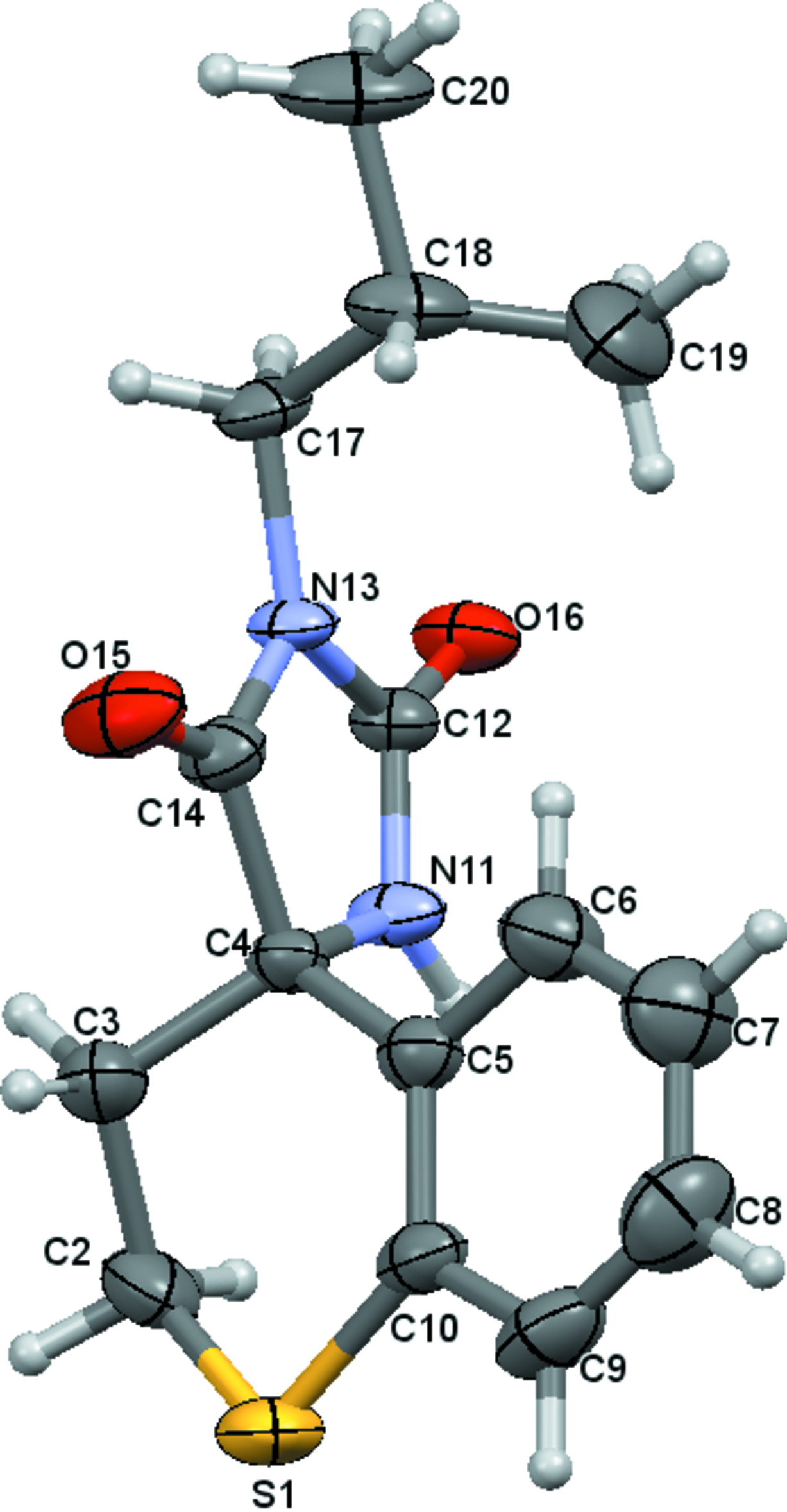

Supplement: Supplementary file 4 [file e-70-o1043-fig1.tif]

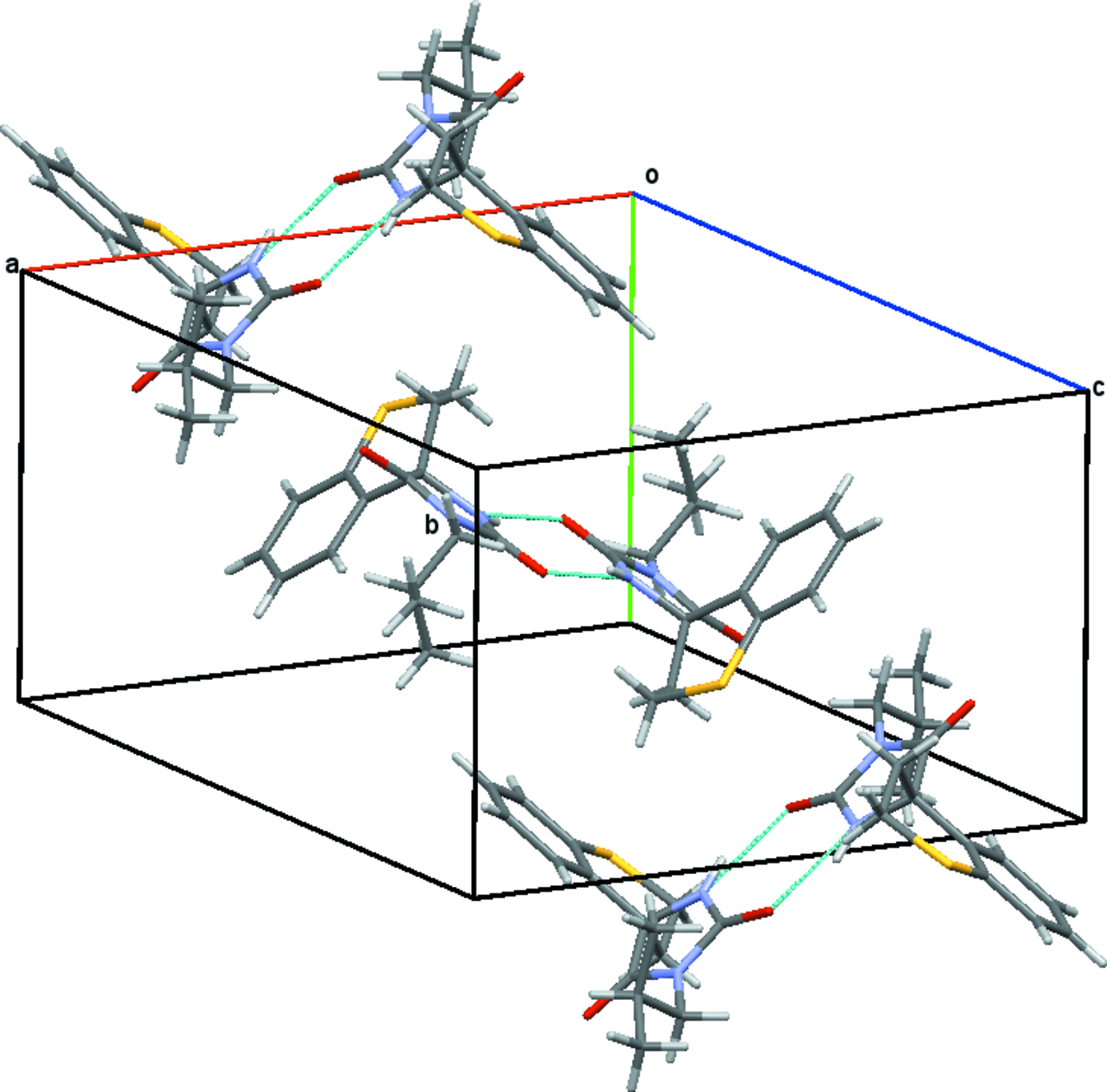

Supplement: Supplementary file 5 [file e-70-o1043-fig2.tif]
